# Ocular Cat Bite Injury: A Case Report and Literature Review

**DOI:** 10.7759/cureus.24636

**Published:** 2022-05-01

**Authors:** Ahmad-Marwan Abdul Aziz, Abdul-Salim Ismail, Azhany Yaakub

**Affiliations:** 1 Department of Ophthalmology and Visual Science, School of Medical Sciences, Universiti Sains Malaysia, Kubang Kerian, MYS; 2 Department of Ophthalmology, Hospital Pulau Pinang, Georgetown, MYS

**Keywords:** prolapse uvea tissue, intravitreal injections, ocular trauma, sclera puncture, cat-bite

## Abstract

Ocular injury related to cat bites is rare, and no proper guidelines have been suggested for the treatment of such injuries. We report a rare case of zone II penetrating ocular injury secondary to a cat bite. A 49-year-old lady presented with left eye pain following a cat bite over the left eye, which occurred four hours prior to the presentation. Immediate primary closure with intravitreal antibiotic injections was given. Systemic and topical antibiotic treatments were administered. Presenting visual acuity was hand motion, which improved to 20/20 at six months follow-up. Throughout this period, there were no signs of endophthalmitis. Prompt and effective antibiotic administration with early surgical intervention contributed to a good visual prognosis in this case.

## Introduction

The majority of the households worldwide have pets as part of the family. In Asia, out of one-third of the population, 32% confirmed that dogs are man’s best friends, while cats followed closely at 26% [1]. Contrary to this, Malaysia and Indonesia recorded cats having the most ownership, 47% and 34%, respectively, outnumbering dog ownerships at 10% and 20%, respectively [1]. Globally, among the injuries related to animal bites, 2%-50% are from cat bites, whereas in terms of incidence, they are usually second to dog bites [2]. The incidence of cat-related injuries is 18 per 100,000 population in Italy, while in the United States of America, every year, it is estimated as 400,000 cat bites and 66,000 visits to hospital emergency departments [2].

Microorganisms such as *Pasteurella*, *Staphylococcus*, *Bartonella*, and *Streptococcus *species are the common resident flora of domestic cats [3]. The teeth of a cat are pointed and sharp and can cause deep wounds. Following a cat bite, the affected area usually closes rapidly over the bite, which may trap microorganisms. Thus, these bites from cats develop infections more frequently than the bites from dogs [4]. Cat bite may cause visual threatening infection such as cat-scratch disease, which is caused by the bacterium *Bartonella henselae*, a gram-negative rod. Intraocular changes include intermediate uveitis, subretinal lesions, retinitis, neuroretinitis, inflammatory masses, and angiomatous lesions. A cat bite can also infect a person with pasteurellosis, caused by bacterial *Pasteurella multocida*, an oxidase-positive, aerobic, gram-negative coccobacillus. A part of normal bacterium lives in the mouths of a healthy cat. Up to 40% of patients with bite wound infections due to *P. multocida* may develop septic arthritis, abscess, osteomyelitis, or tenosynovitis.

There is no proper guideline for the management of cat bites as compared to dog bites. Although cat bites are more often considered minor injuries, they can result in serious infections. Thus, we would like to take this opportunity to elaborate more on the cases of cat bites, with a case report of our own experience in its management and a comparison of cases to other regions locally and globally. While ocular injury related to cats is rare, to date, 10 cases of cat-bite- or cat-scratch-related endophthalmitis have been reported [5-14]. All of the cases reported are associated with endophthalmitis. The most common organism isolated was *P. multocida* in 54.5% of cases.

In this study, we will share our experience in successfully treating a penetrating ocular injury following a cat bite. We will also discuss the similarities and differences of previously reported cases related to cat bites and cat scratches. The injuries involving the open globe will be divided into three zones based on the location of the injuries, namely zone I in which the opening of the globe is isolated to the cornea or corneoscleral limbus, zone II which involves the anterior 5 mm of the sclera, and zone III in which the injuries are located more than 5 mm posterior to corneoscleral limbus [15].

## Case presentation

**Figure 1:**
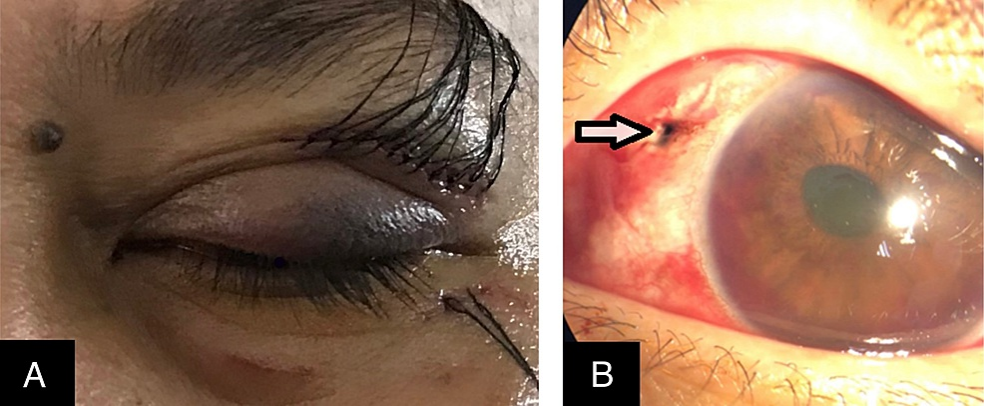
Left eye on presentation (A) Laceration wound over the left eyelid. (B) The puncture wound at 10 o’clock is shown by the arrow. The image shows hyphema inferiorly in the anterior chamber.

A 49-year-old lady alleged a stray-cat bite over the left eye four hours prior to the presentation. Following that, the patient developed a left facial bleed and pain, associated with reduced vision. She sustained a left upper lid partial-thickness laceration wound, as seen in Figure 1, Panel A. In the left eye, the presenting visual acuity was hand motion. There was no relative afferent pupillary defect (RAPD), which was examined via the reverse method due to the left eye's eccentric pupil. There was a subconjunctival hemorrhage at the inferior and nasal quadrants; a puncture wound was seen at 10 o’clock about 4 mm from the limbus, where blackish choroidal tissue was noted. The pupil was eccentric 5 mm and peaked at 10 o’clock. There was a fluidic hyphema with a 1-mm hyphema level in the anterior chamber. In the right eye, the vision was 20/20 with normal anterior and posterior segment examination.

The patient was given an intramuscular antitetanus injection and started on intravenous Ciprofloxacin 400 mg twice a day, and the case was booked for emergency surgery under general anesthesia. Left eye wound exploration, uveal tissue reposition, conjunctiva scleral laceration, and wound toilet and suturing (T&S) were done on the same day. Intraoperatively, as seen in Figure 1 (Panel B), a left eye conjunctiva scleral puncture wound at 10 o’clock was noted at 4.5 mm from the limbus at the superonasal quadrant, zone II injury. Exposed uveal tissue was trimmed, and the remaining was reposited. The anterior chamber was washed out and reformed. The phakic lens was stable with no anterior capsule breach. A vitreous tap was obtained and sent for culture and sensitivity. Prophylactic intravitreal antibiotics, namely Ceftazidime 2 mg/0.1 mL and Vancomycin 2 mg/0.1 mL, were injected.

**Figure 2:**
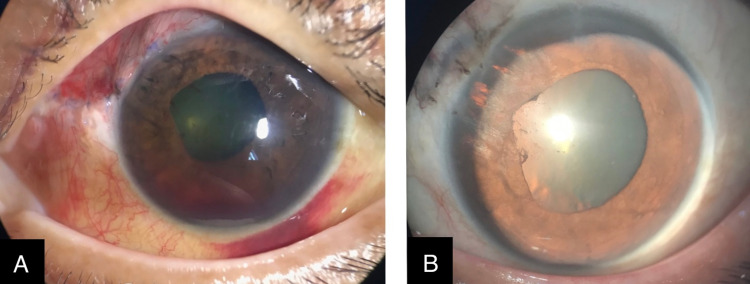
Left eye post-surgery (A) Day 1 post-surgery. (B) Month 1 post-surgery.

Postoperatively, the patient completed the three-day course of intravenous Ciprofloxacin. Then, the topical Ciprofloxacin 0.3% was started every two hours over the left eye, while topical prednisolone acetate 1% for every two hours was also added after the fungal infection was ruled out. Daily monitoring in the ward showed contracting subconjunctival hemorrhage and hyphema, stable intraocular pressure (IOP), and normal fundus examination. There were no signs of infection throughout the admission, as seen in Figure 2 (Panel A). The patient was discharged on day seven of admission with vision 20/200 over the left eye and no sign of endophthalmitis.

Vitreous samples sent for culture and sensitivity showed no growth. The patient responded well with systemic Ciprofloxacin, topical Ciprofloxacin, and topical steroids. An outpatient review one week later showed that at 14 days post-operation, her vision improved to 20/120. At one-month post-surgery as seen in Figure 2 (Panel B), the vision was 20/40 with iris atrophy visible at the superonasal quadrant. The patient was able to gain a vision of 20/30 at two months post-surgery, and she maintained a good vision of 20/20 at six months post-surgery with a clear lens, normal fundus examination, and no signs of endophthalmitis.

## Discussion

According to the management of periocular and adnexal trauma due to animal bites, it is important to ascertain if the animal was rabid or not. It is also important to check if the bite was provoked or unprovoked. In such trauma, the aims of treatment are to prevent wound infection by adequate wound toilet, rabies prophylaxis in case of dog bites, wound exploration, and surgical reconstruction of normal anatomy, with the greatest restoration of function [16,17]. With the variety of resident flora in the oral cavity of animals, wound infection can easily occur. Talan et al. reported in a prospective study that an average of five species of organisms were found in dog and cat bites [18]. The most common organism isolates reported were *Pasteurella*, *Streptococci*, *Staphylococci*, *Corynebacterium*, *Moraxella*, and *Neisseria*. This underscores the importance of antimicrobial prophylaxis.

Since 1973, 10 cases of ocular injury related to cat scratches or cat bites were reported. All 10 cases are associated with endophthalmitis as summarized in Table 1, in which a total of 11 cases including our case report are summarized. The mean age is 19.6 years, ranging from one year old to 51 years old; 64% of cases are female (seven cases). The presenting onset post-injury is not available for three cases. Patients presented post-injury at the earliest onset of four hours to four days post-injury. Both the earliest four hours and the latest four days of presentation gain a final vision of 20/20; thus, we observe that presenting onset does not necessarily contribute to a good visual outcome. We observe that patients with injury at zone I and zone II mostly have a good final visual outcome, that is, out of six patients with zone I and zone II injuries collectively, five patients (83.3%) gain vision 20/20 to 20/40. The remaining patient with zone I injury has persistent retinal detachment, while patients with zone III injury generally have poor final visual outcomes.

**Table 1:** Case report involving ocular injury in relation to cat bites or cat scratch OD: Right eye; OS: Left eye; SN: Superonasal; IN: Inferonasal; IT: Inferotemporal; MR: Medial rectus; LP: Light perception; HM: Hand motion; RD: Retinal detachment.

Cases	Age/Sex	Presenting Onset	Eye	Nature (Scratch/Bite); Location; Zone	Organism	Pars Plana Vitrectomy	Intravitreal Antibiotics	Presenting Vision	Final Vision
Galloway et al., 1973 [5]	11, F	Same day	OD	Scratch; IT quadrant; Zone III	Pasteurella septica	-	-	20/200	Evisceration
Puliafito et al., 1982 [6]	12, M	2 days	N/A	N/A; Cornea laceration; Zone I	Pasteurella multocida	-	-	LP	20/40
Weber et al., 1984 [7]	10, M	N/A	OS	Scratch; Cornea; Zone I	Pasteurella multocida	+	-	N/A	20/30
Yokoyama et al., 1987 [8]	51, M	N/A	OS	Bite; Insertion of MR; Zone III	Pasteurella multocida	+	-	LP	20/400 (RD)
Vartian et al., 1989 [9]	8, F	Same day	OD	Scratch; Sclera; N/A (vitreous prolapse)	Pasteurella multocida, Neisseria animaloris, Neisseria zoodegmatis	+	+	N/A	LP
Zimmer-Galler et al., 1996 [10]	8, F	N/A	OS	Bite; Cornea; Zone I	Capnocytophaga canimorsus	+	+	N/A	LP (persistent RD)
Doi et al., 1999 [11]	23, F	3 days	OS	Scratch; IT quadrant; Zone III	Pseudomonas aeruginosa	+	-	20/250	20/200 (RD)
Iwasaki et al., 2004 [12]	1, M	1 day	OD	Bite; Sclera; N/A	Pasteurella multocida	-	+	N/A	N/A
Mochizuki et al., 2020 [13]	10, F	18 hours	OD	Scratch; Sclera; Zone II	Pasteurella multocida	+	+	20/40	20/20
Kishore et al., 2020 [14]	33, F	4 days	OD	Bite; Sclera IN quadrant; Zone II	Alpha hemolytic streptococcus	+	+	HM	20/20
Ahmad-Marwan, 2020	49, F	4 hours	OS	Bite; Sclera SN quadrant; Zone II	No organism isolated	-	+	HM	20/20

A majority of the cases (54.5%) isolated *P. multocida* with a variable final visual outcome due to different sites of injury involvement. Other organisms include *Pasteurella septica, Neisseria animaloris, Neisseria zoodegmatis, Capnocytophaga canimorsus, Pseudomonas aeruginosa, Alpha hemolytic streptococcus*, which are all isolated in one case each.

Concerning our patient who presented at the earliest onset of four hours post-trauma, an immediate intervention was possible to be carried out. There were no signs of infections and no sign of endophthalmitis. The patient also had a good visual recovery. This is most likely due to early presentation, prompt treatment, and immediate prophylaxis intravitreal antibiotic injections. Other possible contribution is negative culture isolated from the vitreous sample.

Given the growing pet ownerships of cats, the awareness of ocular injury is important. Even if the patients are not pet owners, there are also many opportunities to touch pet animals. Thus, we must emphasize the importance of preventing direct injury from animals as well as taking care of infection controls for resident bacteria from pet animals to prevent future endophthalmitis following intraocular surgery.

## Conclusions

We presented a case of zone II ocular injury as a result of a cat bite. The excellent outcome for this patient is very encouraging. Cat bites potentially cause a debilitating outcome. However, prompt antibiotic treatment and effective surgical intervention provide a good visual prognosis.
